# Understanding Inequalities in Adult Social Care in Wales: Protocol for the CARE Lab Linked Administrative Data Study

**DOI:** 10.23889/ijpds.v11i1.3393

**Published:** 2026-08-03

**Authors:** Fiona Victoria Lugg-Widger, Ashley Akbari, Rebecca Cannings-John, Oliver S. Cumming, Matthew Curds, Miranda Evans, José-Luis Fernandez, Henrietta Graham, Mala Mann, Melissa Meindl, Abigail Palmer, Lisa Trigg, Nell Warner, Paul Willis, Julie Wych, Simone Willis, Jonathan Scourfield

**Affiliations:** 1 Centre for Trials Research, Cardiff University, Wales; 2 Population Data Science, Faculty of Medicine, Health & Life Science, Swansea University, Wales; 3 Knowledge and Analytical Services, Welsh Government, Wales; 4 Disability Wales, Maindy Road, Cardiff, Wales; 5 Care Policy and Evaluation Centre, London School of Economics and Political Science, London, UK; 6 Specialist Unit for Review Evidence, Cardiff University, Wales; 7 Division of Population Medicine, School of Medicine, Cardiff University, Wales; 8 Centre for Adult Social Care Research (CARE), School of Social Sciences, Cardiff University, Wales; 9 Social Care Wales, Wood Street, Cardiff, Wales; 10 Children’s Social Care Research and Development Centre (CASCADE), School of Social Sciences, Cardiff University, Wales

**Keywords:** adult social care, linked data, TRE (Trusted Research Environment), inequalities, national level, wales

## Abstract

**Introduction:**

Adult social care in the UK faces increasing demand and persistent inequalities in terms of access and care quality, yet national-level understanding remains limited. The CARE Lab study aims to address these gaps using newly available individual-level routine administrative data for the whole of Wales from the Adults Receiving Care and Support (ARCS) census. This study will explore patterns of care provision, transitions from children’s to adult services, and socio-demographic disparities, using linked data to inform service planning and policy.

**Methods:**

and analysis This quantitatively-led mixed-methods study comprises five research questions. Quantitative analysis will use ARCS census data, both standalone and linked to health, education, and social care datasets within the Secure Anonymised Information Linkage (SAIL) Databank. Qualitative interviews with people receiving care and support, carers, and professionals will contextualise findings. Key research questions address care patterns, demographic comparisons, regional variation, transitions from child to adult care, and the feasibility of evaluating care models using linked data. Statistical analyses will include descriptive and inferential statistics, propensity score matching, and there will be thematic analysis of qualitative data.

**Ethics:**

Ethical approval has been obtained from Cardiff University. Data access approvals will be sought from Welsh Government, SAIL, and the Office for National Statistics. Dissemination will occur through peer-reviewed publications, policy briefings, accessible multimedia outputs, and stakeholder engagement via an action group. The study will also produce a research-ready data asset and recommendations for future data infrastructure development across the UK.

## Strengths and Limitations of this Study

- The study uses the first national census of adults receiving care and support in Wales, enabling population-scale analysis across all local authorities.
- A mixed-methods design combines quantitative data with qualitative insights from people receiving care and support, carers, and professionals to provide a deeper understanding of care experiences.
- Access to the SAIL Databank, and twenty-five anonymised, routinely collected datasets allows for individual-level linkage across health, education, and social care domains.
- ARCS data excludes individuals whose care is not arranged through local authorities, potentially underrepresenting certain groups and limiting generalisability.
- Minimal nature of the dataset, few variables and limited detail for those not active on census date.
- Linkage rates are not yet available which may impact analysis and interpretation.

## Introduction

Adult social care in the United Kingdom (UK) faces persistent and growing challenges within a mixed economy of provision, including high staff turnover, increasingly complex care needs, and a projected rise in demand (in England) of 41-49% by 2038 [1]. There are inequalities in access to social care and experience of care, by age, gender, ethnicity and geographical location [2–5]. Despite this, there remains a limited national understanding of adult social care provision, particularly regarding local and regional variation, socio-demographic inequalities [6–8], gaps in support [9] and the long-term impact of austerity [10]. Notably, there is no UK evidence at a population level on transitions from children’s to adult social care, representing a significant gap in the life-course perspective of care provision.

While research in adult social care is expanding, methodological limitations persist. Cross-sectional surveys are prone to response bias, and longitudinal studies are limited by attrition bias, resulting in under-representation of certain demographic groups and care needs. In contrast, routinely-collected administrative data offer near-complete coverage of individuals accessing publicly funded services, with the potential for linkage across sectors. These data can yield powerful insights, such as comparing health outcomes across different care pathways. Secure data linkage within Trusted Research Environments (TREs) is central to the UK’s vision for data-driven public service improvement [11, 12], although implementation remains in early stages. Social care is a devolved matter in the UK. Only recently have the four UK nations begun collecting nationally standardised, individual-level data on adults receiving social care [13, 14]. In Wales, local authorities (LAs) submitted data for the Adults Receiving Care and Support (ARCS) census for the first time in 2024.

In children’s social care, national-level administrative data research, including, more recently, data linkage, has already driven impactful research and policy change [15–18]. In adult social care, Wales has made considerable progress with successful linkages between care home addresses, Care and Repair services, homecare worker registration and health data [19–21]. However, the field remains underdeveloped, in part due to the absence of a national care population census. A recent scoping review [22] identified only 12 primary studies from 2012-2023 that linked adult social care data to other research or administrative data sources across the UK. All studies included within-nation linkage, primarily to health data (primary care, secondary care, prescriptions), with no examples of cross-nation linkage. Family carers (unpaid carers) were not present in any of these studies despite search terms specifically included to identify this group.

### Study Aim

This study aims to advance the understanding of the adult population receiving care and support via LAs with the goal of informing service planning, evaluation, and targeted strategies to reduce inequalities. In doing so, it will demonstrate the value (and limitations) of linked administrative data for adult social care research.

## Methods and Analysis

### Research Questions

Through our quantitative analysis, we will answer the following research questions (RQs):

- RQ1: What type of care and support are adults in Wales receiving? How does this vary by impairment/condition, socio-demographic characteristics, caring responsibilities, and LA?
- RQ2: What are the characteristics of adults in Wales receiving care and support compared with the rest of the population? (sex at birth, gender identity, sexuality, age, ethnic group, religion, household composition, language preference, area-level deprivation). How does this vary by impairment/condition, and type of care and support?
- RQ3: To what extent does social care support vary across areas?
- RQ4: What are the characteristics of children who transition to adult social care services compared with those who do not?
- RQ5: To what extent can the social care system be evaluated using individual-level, linked administrative care data?

Through our qualitative research we will understand the experience of how social care resources are distributed from the perspective of practitioners, people receiving care and support, and family carers.

### Study Design

This quantitatively-led mixed methods study combines population-level routine data linkage and qualitative interviews to contextualise and interpret findings. Outcomes are summarised below and in Table 1. A rapid review will inform RQ1-3 and qualitative enquiry by synthesising existing evidence addressing the question: *How do individuals with different types of needs, from various geographical areas, socio-economic statuses and protected characteristic groups access and experience adult social care services?* [23]. The rapid review will be conducted using accelerated systematic review methods, as described by Mann et al. [24], enabling timely synthesis of relevant knowledge to support the quantitative analysis. The review will follow guidance from the Cochrane Rapid Review Methods Group [25].

**RQ1: What type of care and support are adults in Wales receiving? How does this vary by impairment/condition, socio-demographic characteristics, caring responsibilities, and LA?**

This cross-sectional analysis uses the ARCS census (2023/24), including all adults with a care and support plan. Outcomes include type and duration of support, residence and unpaid care. Analyses will be descriptive across demographic groups, impairment type and local authority. Findings will be interpreted alongside evidence from the rapid review and qualitative interviews.

**RQ2: What are the characteristics of adults in Wales receiving care and support compared with the rest of the population? How does this vary by impairment/condition, and type of care and support?**

Using linked ARCS data (2024/25–2025/26) within the SAIL Databank, adults receiving care will be compared to the wider population. Linked Census and health data will provide detailed socio-demographic and clinical characteristics. Analyses will estimate prevalence and describe differences between groups, informed by the rapid review. Qualitative work will explore barriers and facilitators to accessing care.

**RQ3: To what extent does social care support vary across areas?**

Using the same linked data, variation in care provision will be examined by local authority, deprivation, and rural/urban classification. Analyses will include descriptive comparisons and regression modelling. Findings will be interpreted in the context of existing evidence and explored qualitatively to understand local drivers of variation.

**RQ4: What are the characteristics of children who transition to adult social care services compared with those who do not?**

Linked children’s social care, education, and ARCS data will be used to identify young people aged 16–18 and assess transitions into adult services. Analyses will describe transition rates and compare characteristics between groups. Qualitative interviews will explore experiences of transition.

**RQ5: To what extent can the social care system be evaluated using individual-level, linked administrative care data?**

This feasibility analysis will assess the use of linked data to evaluate care models. Outcomes include mortality, hospital admission, and care home admission. Analyses will use matching and propensity score methods, informed by directed acyclic graphs, to assess feasibility of causal inference.

*Overall Study Structure:*The study runs over 36 months (from April 2025).

- Year 1: Standalone ARCS analysis (RQ1)
- Years 2–3: Linked data analyses (RQ2–5)

Throughout: Iterative qualitative research and stakeholder engagement to support interpretation and impact

### Study Setting

This study is set in Wales, a devolved nation of the United Kingdom with a population of approximately 3.2 million people [26]. Adult social care is delivered through 22 local authorities, which are responsible for assessing need and arranging care and support services for eligible individuals under the Social Services and Well-being (Wales) Act 2014 [27]. Provision includes a mixed economy of care across public, private and third-sector organisations, with variation in service models, availability, and population need across areas.

This study focuses on adults receiving care and support arranged by local authorities, representing the population captured within national administrative data. While this provides near-complete coverage of publicly funded care, individuals who fully self-fund their care without local authority involvement are not included, estimated at around 20% of older adults in England [13].

To examine this population, the study uses the Adults Receiving Care and Support (ARCS) census, first collected nationally in 2024, alongside linked administrative data within the Secure Anonymised Information Linkage (SAIL) Databank.

#### Data Sources

*The Adults Receiving Care and Support (ARCS) Census*: The ARCS census is a national administrative dataset introduced in 2024, capturing all adults who had a local authority-arranged care and support plan during the financial year (01 April to 31 March) [28]. It includes demographic characteristics, impairment/condition, language preference, and core information on care provision (e.g. type of support, care status, safeguarding, and unpaid care) [Appendix 1].

The dataset is structured as an annual census rather than a continuous longitudinal record. Age is recorded within each annual return and reflects age during the reporting year. While individuals can be linked across years, detailed information on care intensity and complete care histories is limited.

*The Secure Anonymised Information Linkage Databank (‘SAIL’)*: From 2024/25 onwards, ARCS data will be linked within the SAIL Databank, the national TRE for Wales, established over 15 years ago. It is a world leader in the field of data linkage, secure infrastructure and access for research purposes, holding ISO27001 accreditation and Digital Economy Act (DEA) accreditation, enabling SAIL to securely link and hold a variety of anonymised, population-scale, individual-level, routinely-collected data sources relating to individuals living in Wales [29].

The first ARCS return (2023/24) is available as a standalone dataset and will be used for descriptive analyses. Subsequent years (2024/25–2025/26) will be used for linked analyses. The CARE Lab study will be the first project to access and analyse these newly linked data.

### Participants

Participants are defined separately for each research question and the qualitative research. **RQ1: Care population (standalone analysis):** Adults receiving care and support in Wales during the census period of 01 April 2023 to 31 March 2024, as recorded in the ARCS dataset, representing the national population receiving local authority care and support.

**RQ2–3 and RQ5: Linked care and comparison populations:** Adults receiving care and support during 2024/25–2025/26 whose records can be successfully linked within the SAIL Databank. Records lacking high-quality linkage or key linkage fields will be excluded. For linked individuals, all available data across relevant datasets will be included. The comparison population will be drawn from the wider adult population of Wales within SAIL. Analyses may be restricted to individuals linked to specific datasets, depending on the research question.

**RQ4: Transition population:** Children receiving care and support between ages 16–18 who are expected to transition to adult social care services, with follow-up into adulthood (to March 2026) to identify transition outcomes.

**Qualitative participants:** Participants will include adults receiving care and support, unpaid carers, and professionals (e.g. social workers, occupational therapists, service managers) across Wales. Sampling will be purposive, informed by emerging findings from RQ1–4, and will aim to capture variation in geography, service context, and participant characteristics.

### Sample Size

**RQ1: Care population (standalone analysis):** We will receive data on the whole population of adults with a care and support plan in Wales, and will contain 60,180 adults [30].

**RQ2–3 and RQ5: Linked care and comparison populations:** Adults receiving care and support during 2024/25 and 2025/26 will be linked to data sources within the SAIL Databank, using two years of ARCS census returns. Records lacking high-quality linkage results or missing key linkage fields will be excluded. For individuals with successful linkage, data will be included for all available years. The expected sample size is approximately 80,000 adults with a care and support plan, based on an estimated 60,180 individuals returned annually and 20,000 new cases each year [28]. The age distribution is skewed towards older adults, with 23% aged 18–54 and the majority aged 65+, particularly 75–84 (24%) and 85+ (32%) [31]. A larger comparison group of the adult population in Wales will also be included, providing ample sample size to estimate prevalence rates with high precision (using 95% confidence intervals (CIs)). Sample size estimates for analysis in RQ5, using propensity score modelling, will be outlined when outcomes are determined and included in the RQ5 statistical analysis plan (SAP).

**RQ4: Transition population:** For the analysis of transitions between children’s and adults’ services the analysis, the estimates are of around 1,500 young people per year leaving children’s services (in receipt of care and support) [32, 33].

**Qualitative research:** We will interview n≈60 individuals; approximately 15 interviews per RQ1-4, consisting of a minimum of five interviews with relevant people with direct experience of receiving social care support (including carers), diverse in terms of protected characteristics (dependent on the research question) plus approximately ten interviews with staff and senior leaders in the sector.

### Data Collection and Management

Data collection, linkage and management procedures are aligned to each research question and summarised below.

**RQ1: What type of care and support are adults in Wales receiving? How does this vary by impairment/condition, socio-demographic characteristics, caring responsibilities, and LA?**

This analysis will use the standalone ARCS dataset for 2023/24. Data will be cleaned and coded using the ARCS data dictionary, in collaboration with Welsh Government where required. Variables will be derived to describe care provision, demographic characteristics, and support arrangements.

Data are stored within the Welsh Government Secure eResearch Platform (WGSeRP) and accessed by accredited researchers. Cleaning and analysis code will be developed in this environment and subsequently transferred to SAIL to support consistency across study phases.

**RQ2 and 3: What are the characteristics of adults in Wales receiving care and support compared with the rest of the population? How does this vary by impairment/condition, and type of care and support? To what extent does social care support vary across areas?**

These analyses will use linked ARCS data (2024/25–2025/26) within the SAIL Databank. Data linkage will be conducted using a standard split-file approach, with Digital Health and Care Wales acting as a trusted third party to assign anonymised linking fields [ALF] [29].

ARCS records successfully matched with an ALF will then be linked to the following data sources [34] within the SAIL Databank TRE. These include:

- **Demographic and population data:** Welsh Demographic Service Dataset (WDSD), Census 2021 (CENS)
- **Health datasets:** Patient Episode Database for Wales (PEDW), Welsh Longitudinal General Practice (WLGP), Emergency Department Data Set (EDDS), Outpatient Database for Wales (OPDW), Critical Care Dataset (CCDS), Intensive Care National Audit & Research Centre (ICNC), Wales Results Reporting Service (WRRS), NHS 111 data (NHSO), COVID-19 vaccination (CVVD) and testing data (CVLF, PATD)
- **Maternity and child health:** Maternity Indicators Dataset (MIDS), National Community Child Health (NCCH), Annual District Birth Extract (ADBE)
- **Social care and education:** Children Receiving Care and Support (CRCS), Looked After Children Wales (LACW), Education Wales (EDUW)
- **Other relevant datasets:** Annual District Death Extract (ADDE), Substance Misuse Data Set (SMDS), and care home address data from Care Inspectorate Wales (CARE)

For both cohorts (adults receiving care and support and the wider population), health conditions and impairments will be derived from electronic health records using established phenotypes from the SAIL concept library [35]. Primary care events and associated diagnoses, medications, referrals and administrative information from WLGP will be identified using published Read codes version 2. Secondary care admissions and associated diagnoses, operations and administrative information will be identified from PEDW, along with emergency department events from EDDS and outpatient appointments from OPDW using International Classification of Diseases version 10 (ICD-10) codes. The SMDS will provide information about whether individuals have used NHS Substance Misuse Services. The WDSD will identify the households and household composition of those receiving care and support using a Residential Anonymous Linking Field [36], a facility in the SAIL Databank which enables individuals to be anonymously linked to those registered with GPs at the same address. The WDSD will also provide information about small area-level deprivation of households as measured by Lower-layer Super Output Area (LSOA) Welsh Index of Multiple Deprivation (WIMD) version 2019 quintile. The CARE data source of care home addresses will similarly anonymously identify those living at a care home address.

**RQ4: What are the characteristics of children who transition to adult social care services compared with those who do not?**

This analysis will build on the linked data infrastructure described above. ARCS data will be linked to children’s social care and education datasets within SAIL to identify young people approaching transition age and track outcomes into adulthood.

Additional derived variables will include indicators of additional learning needs and prior care history. Data preparation and linkage processes will follow those described for RQ2-3.

**RQ5: To what extent can the social care system be evaluated using individual-level, linked administrative care data?**

This analysis will use the linked datasets described above, alongside publicly-available data from Care Inspectorate Wales on provider type, location and size, with additional data on provider sector, which are not publicly available, subject to negotiation of data access. Exposure groups representing different models of care will be derived using ARCS and these external data sources. By ‘care models’, we refer to differences in the types of help provided across areas, including the balance between community-based and residential care, sector provision (private, local authority, charity) and provider characteristics (e.g. size). Outcomes (Table 1) will be identified from linked health and demographic datasets. Data preparation will include construction of analysis-ready cohorts and variables required for matching and causal inference approaches.

**Table 1:** Summary of Research Questions, Populations, and Outcomes

**Research Question**	**Population**	**Outcomes**
**RQ1: What type of care and support are adults in Wales receiving, and how does this vary by impairment/condition, socio-demographic characteristics, caring responsibilities, and LA?**	Adults receiving care and support (ARCS 2023/24)	**All adults:** duration of support (days); incidence of active care/support. **For those with active plans:** type of care and support; residence type; safeguarding; support from an unpaid carer; own caring responsibilities.
**RQ2: What are the characteristics of adults receiving care and support compared with the wider population?**	Adults receiving care and support (ARCS 2024/25 – 2025/26 vs general population (SAIL)	Linkage rate; prevalence of adults in Wales receiving care and support (by socio-demographic and health conditions).
**RQ3: To what extent does social care support vary across areas?**	As per RQ2	Prevalence of adults in Wales receiving care and support by area.
**RQ4: What are the characteristics of children who transition to adult social care services compared with those who do not?**	Young people (16–18) in children’s services	Prevalence of children who transition to adult social care services.
**RQ5: To what extent can the social care system be evaluated using linked data?**	As per RQ2	**Feasibility outcomes**: mortality; care home admission; rates of care and support provision in LAs; time to hospital admission.

#### Qualitative Data Collection

Staff will be invited to take part via LAs and social care organisations. Organisations to approach will be selected based on a principle of best fit with RQs, keeping in mind the relevant user population and types of services offered by the organisation. For each RQ, we aim to recruit representatives from at least two LAs (one rural, one urban). Letters of invitation will be sent to senior leaders and heads of departments across selected organisations. We will also invite senior leaders in organisations to participate in interviews, aiming to assess their strategic thinking on addressing identified trends through the planning, commissioning, and delivery of services. Gatekeepers will be requested to circulate participation invitations to relevant staff. Letters of invitation will be circulated to people with lived experience through user-led groups and third-sector organisations. Members of the Centre for Adult Social Care Research (CARE) public involvement board also represent eight key organisations (e.g. Carers Trust Wales, All-Wales People First) who will be approached to assist with recruitment. Interview schedules will be developed with the CARE Lab Lived-Experience Group. They will explore ongoing barriers and enablers in providing care and support, and, if relevant, perceived differences between LAs or services. We anticipate adopting a semi-structured format using interview schedules developed in consultation with our lived experience advisory group and based on emerging findings from RQ1. Most interviews will run between 30 mins and an hour and be facilitated by telephone or video call via MS Teams. Members of the CARE Lab lived-experience group will participate in some of the interviews, alongside a CARE Lab staff member, following training in qualitative interviewing.

### Statistical Methods

All analyses will be conducted within secure Trusted Research Environments (WGSeRP and SAIL) using SQL, R and Stata (V.18). Across all research questions, standard descriptive statistics will be used to summarise populations and outcomes, including counts, proportions, and presented with 95% confidence intervals where relevant. Analyses will be guided by pre-specified statistical analysis plans [37].

**RQ1: What type of care and support are adults in Wales receiving? How does this vary by impairment/condition, socio-demographic characteristics, caring responsibilities, and LA?**

This cross-sectional analysis will describe the population receiving care and support in 2023/24. Analyses will summarise care provision, demographic characteristics, and support arrangements, including variation by impairment/condition and local authority. Data completeness and consistency across variables will also be assessed. As ARCS represents a population-level dataset, linkage is not required to define the denominator. However, population characteristics will be compared across subsequent ARCS returns to assess consistency and identify potential data quality issues.

**RQ2: What are the characteristics of adults in Wales receiving care and support compared with the rest of the population? How does this vary by impairment/condition, and type of care and support?**

We will report linkage rates of the ARCS data to the SAIL Databank describing characteristics (gender, age, ethnic group, impairment/condition, LA) of individuals not linked (vs linked). To understand which characteristics are associated with receipt of care and support (and type of care), we will describe both cohorts (adults in Wales receiving care and support or not). We will break new ground in reporting on many protected characteristics in relation to social care at a national level. The 2021 Census will be used to derive this information about the individual’s sexuality, gender identity, and religion, information not collected through ARCS or reliably through health or LA records. Individuals with specific health conditions and impairments will be identified from the SAIL Databank sources, and we will report prevalence estimates presented alongside 95% CIs in individuals with and without care and support arranged by their LA. We will describe assumptions made in the data, or any limitations that inform RQ5 for further evaluation.

**RQ3: To what extent does social care support vary across areas?**

We will examine how the prevalence of adults receiving care and support with specific health conditions / impairments varies by areas of Wales based on the LSOA of residence such as WIMD deprivation quintiles and whether this varies by type of care. We will also examine variation in social support by LA, and rural/urban categories. Specific health conditions and impairments will be identified following discussion with lived-experience and action groups but are likely to include dementia and learning disability. Analysis will initially be descriptive, using graphical methods to demonstrate variation, if necessary. We will consider comparing areas (WIMD quintiles, rural vs urban, LA) using appropriate regression models (e.g. Poisson regression with robust (sandwich) variance), to produce crude and adjusted rate ratios. In line with the social model of disability, we acknowledge that health status is not a good proxy for social care need, however ARCS does not yet include data on assessed social need. This preliminary analysis will prepare the ground for future work with a more fine-grained future iteration of ARCS. We also acknowledge that self-funders may not approach LA assessment but go directly to the market. Acknowledging this important limitation, we will assess evidence of an adult social care ‘inverse care law’ (i.e., when less care is provided in more socioeconomically deprived areas).

**RQ4: What are the characteristics of children who transition to adult social care services compared with those who do not?**

We will describe how many children who had aged out of the children’s social care system received adult social care services in 2024-6 and describe by key characteristics (including type of additional learning need and health status). This will be facilitated by linking CRCS, LACW, education (for additional learning needs), and ARCS data. The analysis plan for this RQ will also be informed by the Social Care Wales (SCW) research prioritisation work on transitions [38].

**RQ5: To what extent can the social care system be evaluated using individual-level, linked administrative care data?**

We will develop a directed acyclic graph (DAG) to identify factors associated with exposures and outcomes and assess their availability within the data. Outcomes of interest include destination outcomes (e.g. care home admission) derived from PEDW, WDSD and CARE, while health conditions and impairments will be identified from CCDC, EDDS, ICNC, NHSO, OPDW, PEDW, SMDS and WLGP and area-level balance of care categories and provider types will be identified from Care Inspectorate Wales data.

To examine the effects of exposure on outcomes, we will use matched comparison approaches, including direct matching on key characteristics (e.g. age, sex) and propensity score methods.

Propensity scores will be estimated using logistic regression, incorporating measured confounders identified through the DAG, and used to match exposed and unexposed individuals [39]. Effects will be estimated as differences in outcomes between matched groups.

Both approaches will be assessed in this feasibility study, considering comparator identification, confounder control, and resulting effect estimates. Given the limited follow-up (maximum two years), full outcome evaluation will be constrained. Therefore, this work will focus on the feasibility of evaluating care models using outcomes such as mortality, time to hospitalisation, rates of care and support provision in LAs and care home admission.

Study findings will be reported in accordance with applicable reporting guidelines for observational studies using administrative data (STROBE [40] and RECORD [41]).

**Qualitative analysis:** Interviews will be audio recorded, transcribed, anonymised and thematically analysed. A coding framework will be developed and coding facilitated by NVivo software. The CARE Lab Lived-Experience Group will be invited to share in data interpretation and feedback on emerging themes.

### Capacity Building

Two separately funded PhD studentships will extend the CARE Lab programme. One will focus on adult safeguarding, using ARCS data to examine inequalities in Care and Support Protection Plans by demographic and contextual factors, with subsequent qualitative work exploring potential barriers to safeguarding. A second studentship, commencing in 2026, will be aligned with CARE Lab’s objectives and the available linked data.

### Public Involvement

A CARE Lab Lived Experience Group has been established to advise on all aspects of the study. The group includes adults with experience of receiving care and support, unpaid carers, and members from diverse backgrounds. It meets three times per year and includes representation on the study oversight and action groups. Members were recruited from the CARE Public Involvement Board and the user-led organisations represented on this board, through Disability Wales’s membership and Age Cymru. To ensure ethnic diversity, the group was advertised to relevant groups representing minoritised communities. The Group is facilitated by (author) ME from Disability Wales.

The terms ‘impairment’ and ‘condition’ mentioned in this manuscript reflect classifications used within administrative datasets; we acknowledge that these may not align with individuals’ own identities, and the Group will inform how findings are interpreted and communicated to ensure appropriate and respectful framing.

The group will inform study materials, interpretation of findings, dissemination, and the language used to describe care, impairment and condition categories. Members will also contribute to selected qualitative interviews following appropriate training.

## Ethics and Dissemination

An ethical self-assessment has been submitted to the School of Social Sciences Research Ethics Committee for RQ1-5. Ethical approval has been obtained from Cardiff University school of social sciences ethics panel for the qualitative work [REF:822]. Approval for the standalone ARCS dataset was granted by Welsh Government on 02 June 2025. SAIL Information Governance Review Panel approval has been granted, with access to linked data expected from June 2026. Further approvals for linkage to ONS Census, education, children’s social care and ARCS data will be sought.

A study oversight group will provide scientific and policy oversight. An action group, including people with lived experience, policy and practice stakeholders, will support interpretation and dissemination.

Findings will be shared through peer-reviewed publications, policy and practice briefings, accessible infographics and videos, professional networks, and conferences. The study will also produce recommendations for future ARCS development, a data resource profile, and research-ready datasets, metadata, code and documentation to support reproducibility.

### Horizon Scanning

CARE Lab will identify limitations in the ARCS dataset and generate recommendations to strengthen adult social care data infrastructure. This will include consideration of coverage, classification, completeness, and the feasibility of linkage across health, social care and wider administrative data. Key considerations include under-ascertainment of care packages (e.g. those not active on the census date), limitations in impairment categorisation (e.g. broad neurodevelopmental categories), and gaps in coverage such as joint health and social care packages.

Recommendations will be informed by researchers using the data, the lived experience and action groups, and engagement with Welsh Government through the ARCS Working Group. Consultations with counterparts across the UK will explore readiness for similar linked adult social care data initiatives in other nations.

## Supplementary Files

Supplementary Appendix 1

## Abbreviations

ADBE:	Annual District Birth Extract
ADDE:	Annual District Death Extract
ADR:	Administrative Data Research
ARCS:	Adults Receiving Care and Support census
CARE:	Care home addresses from Care Inspectorate Wales
CARE	
Centre:	Centre for Adult Social Care Research
CCDS:	Critical Care Dataset
CENS:	The Office for National Statistics (ONS) Census 2021
CI:	Confidence Interval
CINW:	Children in Need Wales.
CRCS:	Children Receiving Care and Support
CVLF:	COVID-19 Lateral Flow Testing Data
CVVD:	COVID Vaccine Data
DAG:	Directed Acyclic Graph
DEA:	Digital Economy Act
EDAD:	Education Attendance Data
EDDS:	Emergency Department Data Set
EDUW:	Education Wales
ICD-10:	International Classification of Diseases version 10
ICNC:	Intensive Care National Audit & Research Centre.
IGRP:	Information Governance Review Panel
LA:	Local Authority
LACW:	Looked After Children Wales
LSOA:	Lower-layer Super Output Area
MIDS:	Maternity Indicators Dataset
NCCH:	National Community Child Health
NHSO:	NHS 111 Call data
ONS:	Office for National Statistics
OPDW:	Outpatient Database for Wales
PATD:	COVID-19 Pathology Test results
PEDW:	Patient Episode Database for Wales
RQ:	Research Question
SAIL:	The Secure Anonymised Information Linkage Databank
SAP:	Statistical Analysis Plan
SCW:	Social Care Wales
SMDS:	Substance Misuse Data Set
SQL:	Structured Query Language
TRE:	Trusted Research Environments
UK:	United Kingdom
WDDS:	Wales Dispensing Data Set
WDSD:	Welsh Demographic Service Dataset
WG:	Welsh Government
WGSeRP:	Welsh Government Secure eResearch Platform
WIMD:	Welsh Index of Multiple Deprivation
WLGP:	Welsh Longitudinal General Practice
WRRS:	Wales Results Reporting Service
