# Supplementary material for: Understanding Inequalities in Adult Social Care in Wales: Protocol for the CARE Lab Linked Administrative Data Study
Source: Int J Popul Data Sci. 2026 Aug 3;11(1):3393. doi: 10.23889/ijpds.v11i1.3393 (PMC13492271; doi:10.23889/ijpds.v11i1.3393)
Supplement: Supplementary file 1 [file ijpds-11-3393-s001.pdf]

Appendix 1: ARCS Data items, descriptors and categories

Category	ARCS Census Data 2023/24		Changes over years
	Question text	Answer options	
**Local authority code**	The questions below are to be completed for all adults who have had a care and support plan at any point in the financial year (2023-24).	Isle of Anglesey; Gwynedd; Conwy; Denbighshire; Flintshire; Wrexham; Powys; Ceredigion; Pembrokeshire; Carmarthenshire; Swansea; Neath Port Talbot; Bridgend; Vale of Glamorgan; Rhondda Cynon Taf; Merthyr Tydfil; Caerphilly; Blaenau Gwent; Torfaen; Monmouthshire; Newport; Cardiff	
**Local authority identifier**	This must be a unique ID for each adult, and it should be retained from year to year.	freetext	
**surname**	–	–	*** Collected Y2 onwards**
**First name(s)**	–	–	*** Collected Y2 onwards**
**Adult's home postcode**	–	–	*** Collected Y2 onwards**
**Date of birth**	The date of birth should be provided in the format DD/MM/YYYY. If the exact date of birth is not known, record an approximate date of birth based on the adult's estimated age at date of referral.	DD/MM/YYYY	
**Gender**	Gender is how the individual identifies themselves, not sex at birth.	1 Male<br>2 Female<br>3 Non-Binary<br>4 Not disclosed or unknown	**Changed 'Non-binary' to 'Other' Y2 onwards**
**Ethnicity**	Ethnicity should be determined by first asking the adult about their ethnic identity. If they are unable, ask their primary carer.	WHTE1 - Gypsy or Irish Traveller<br>WHTE2 - Roma<br>WHTE3 – Any other White background<br>MIXD1 - White and Black Caribbean<br>MIXD2 - White and Black African<br>MIXD3 - White and Asian<br>MIXD4 - Any other mixed background / multiple ethnic background<br>ASAB1 - Indian<br>ASAB2 - Pakistani<br>ASAB3 - Bangladeshi<br>ASAB4 - Chinese<br>ASAB5 - Any other Asian background<br>BBAC1 - Caribbean<br>BBAC2 - African<br>BBAC3 - Any other Black background<br>OOTH1 - Arab<br>OOTH2 - Any other ethnic group<br>UNKN1 – Unknown	**Clarified Y2 onwards: Any other White background (including English, Welsh, Scottish, Northern Irish or British)**
**Disability**	Record whether an adult has a disability on 31 March 2024 (or for closed cases at the time the adult's care and support plan was closed).	1 / 0	

Continued

Appendix 1: Continued

Category	ARCS Census Data 2023/24		Changes over years
	**Question text**	**Answer options**	
**Disability | Impairment**	Disability – None<br>Disability – Visual Impairment<br>Disability – Hearing Impairment<br>Disability – Physical Impairment<br>Disability – Mental illness<br>Disability – Dementia<br>Disability – Neurodevelopmental condition<br>Disability – Other (not listed)<br>other	yes/no<br>yes/no<br>yes/no<br>yes/no<br>yes/no<br>yes/no<br>yes/no<br>yes/no<br>freetext	**'Disability' changed to 'Impairment' Y2 onwards Additional question added Y3 onwards: Does the adult have a disability? Yes/No**
**Language**	Record the adult's preferred language; this is the language the adult prefers to use to communicate with others. If there are differences between the preferred written or spoken language, use the language in which care and support is provided.	*See language Code list	
**Care support plan**	Care and support plan active on 31 March	Enter 1 if the care and support plan was active on 31 March 2024<br>Enter 0 if the care and support plan was not active on 31 March 2024	
	For those adults who have 0 for Care and Support plan active on 31 March recorded, please enter the date the care and support plan was closed. If the plan is still active on 31 March, then leave blank.	DD/MM/YYYY	**Clarified details related to closure in explanatory text Y2 onwards**
	Reason for closure For those adults whose care and support plans closed during the year and have been recorded above, please enter the most appropriate reason the care and support plan was closed from the list below: Only one category from the above may be chosen. This cannot be left blank for those adults who do not have an active care and support plan on 31 March 2024.	1 – Service completed<br>2 – Transferred/moved to another local authority<br>3 – Step down to preventative services (and no longer requires ongoing care and support)<br>4 – Moved to health service (e.g., Continuing Healthcare)<br>5 – Refused service<br>6 – Died<br>7 – Other	
**Adult Safeguarding**	For adults who have started formal safeguarding procedures.	Enter 1 if the adult had enquiries in the collection year where it was determined additional action was required and resulting in a care and support protection plan<br>Enter 0 if the adult did not have enquiries in the collection year where it was determined additional action was required and resulting in a care and support protection plan	

Continued

Appendix 1: Continued

Category	ARCS Census Data 2023/24		Changes over years
	**Question text**	**Answer options**	
**Category of safeguarding**	This is intended to record safeguarding categories for all adults on undergoing safeguarding, regardless of when enquiries were made. Record the category or categories of abuse and neglect recorded on the adult's Care and Support Protection Plan (where details of abuse or neglect were included). The category used should indicate the primary concern at the point additional action was identified. More than one category may apply.	• Neglect<br>• Physical<br>• Sexual<br>• Financial	**Clarified requirement to indicate 1 / 0 for each option Y2 onwards**
	Note that emotional / psychological abuse is considered always to occur if one of the other types of abuse occurs. Therefore, it is only recorded here if it occurs in isolation without other types of abuse. If 1 is entered for "emotional/psychological (only)", all other categories should be recorded as 0.	• Emotional/Psychological (only)	
**Residence**	The type of residence for the individual on 31 March should be recorded. Please select the type of accommodation the adult is in on 31 March.	Enter 1 if the adult is in their own home (i.e., residing in the community). This includes where the adult is in a residential care home or supported accommodation on a short-term placement or respite care – but would typically reside at home. This applies to both local authority-provided and private care homes/supported accommodation.<br>Enter 2 if the adult is living in a residential care home or supported accommodation on a long-term placement. As above, this applies to both local authority-provided and private care homes/supported accommodation.<br>Enter 3 if the adult is currently being held in the secure estate. Only select 3 if the adult is being held for a term of more than three months.	**Clarified: Adults in an Adult Placement Scheme (Shared Lives) should be recorded here. Y3 onwards.**
**Current Safeguarding**	Enter 1 if the adult had an active care and support protection plan for adults on 31 March where the local authority considers it necessary to protect an adult from abuse or neglect or a risk of abuse or neglect.	Enter 0 if the adult did not have an active care and support protection plan for adults on 31 March where the local authority considers it necessary to protect an adult from abuse or neglect or a risk of abuse or neglect.	

Continued

Appendix 1: Continued

Category	ARCS Census Data 2023/24		Changes over years
	Question text	Answer options	
**Types of care and support**	Please select what types of care and support the individual is currently receiving (i.e., on 31 March). An individual may be provided with more than one type of care and support, please select all types that the individual is receiving.	• Domiciliary care<br>• Day care<br>• Respite care<br>• Short breaks<br>• Adult placements<br>• Supported accommodation/supported living<br>• Direct payments<br>• Adult care homes (without nursing)<br>• Adult care homes with nursing<br>• Telecare	**Combined Respite care / Short breaks / Replacement care Y2 onwards**<br>**Telecare removed Y2 onwards**<br>**Reablement added Y3**
**Has a known carer**	Whether the person receives support from an unpaid (informal) carer gives a holistic view of a person's support package. It is recognised that there can be multiple unpaid (informal) carers known to the client and actively providing support. For the purposes of the collection, a value of 'Yes' would indicate that at least one carer is known to the client on 31 March.	Enter 1 if the individual has one or more known carer. Enter 0 if the individual has no identified carer.	*** Added Enter 88 if this information is not known Y3 onwards**
**Caring responsibilities**	Please record whether the adult also has caring responsibilities. A carer is someone who provides or intends to provide care for an adult or disabled child.	Enter 0 If the adult has no caring responsibilities on 31 March.<br>Enter 1 if the adult has known caring responsibilities on 31 March and receives known, ongoing support as a carer.<br>Enter 2 if the adult has known caring responsibilities on 31 March but it is not known whether they receive ongoing support as a carer.<br>Enter 88 if this information is not known.	
**Events**	As part of the individual's ongoing care record, the number of assessments, re-assessments and reviews should be recorded. Please indicate how many of each of these events have occurred in the reporting year for the adult receiving care and support.	• Number of assessments (including any specialist assessments)<br>• Number of re-assessments (including any specialist assessments)<br>• Number of reviews	

*Language code list: English; Welsh/Cymraeg; Acholi; Adangme; Afar-Saho; Afrikaans; Akan/Twi-Fante; Albanian/Shqip; Alur; Amharic; Arabic; Armenian; Assamese; Assyrian/Aramaic; Anyi-Baule; Aymara; Azeri; Bamileke (Any); Balochi; Beja/Bedawi; Belarusian; Bemba; Bhojpuri; Bikol; Bislama; Balti Tibetan; Burmese/Myanma; Bengali; British Sign Language; Basque/Euskara; Bulgarian; Cambodian/Khmer; Catalan; Caribbean Creole English; Caribbean Creole French; Chaga; Chattisgarhi/Khatahi; Chechen; Chinese; Chokwe; Cornish; Chitrali/Khowar; Chichewa/Nyanja; Czech; Danish; Dagaare; Dagbane; Dinka/Jieng; Dutch/Flemish; Dzongkha/Bhutanese; Ebira; Edo/Bini; Efik-Ibibio; Esan/Ishan; Estonian; Ewe; Ewondo; Fang; Fijian; Finnish; Fon; French; Fula/Fulfulde-Pulaar; Ga; Gaelic/Irish; Gaelic (Scotland); Georgian; German; Gogo/Chigogo; Kikuyu/Gikuyu; Galician/Galego; Greek; Guarani; Gujarati; Gurenne/Frafra; Gurma; Hausa; Hindko; Hebrew; Herero; Hungarian; Hindi; Iban; Idoma; Igala; Igbo; Ijo (Any); Ilokano; Itsekiri; Icelandic; Italian; Javanese; Jinghpaw/Kachin; Japanese; Kikamba; Kannada; Karen (Any); Kashmiri; Kanuri; Kazakh; Katchi; Kirghiz/Kyrgyz; Khasi; Kihaya/Luziba; Kinyarwanda; Kirundi; Kisi (West Africa); Kalenjin; Kimbundu; Kimeru; Konkani; Kinyakyusa-Ngonde; Kikongo; Korean; Kpelle; Krio; Kru (Any); Kisii/Ekegusii (Kenya); Kisukuma; Kurdish; Lao; Luba; Luganda; Lugbara; Lugisu/Lumasaba; Lingala; Lithuanian; Lango (Uganda); Lozi/Silozi; Lusoga; Latvian; Luxemburgish; Luvale/Luena; Lunda; Luo (Kenya/Tanzania); Luhya (Any); Magahi; Maithili; Makua; Manding/Mandekan; Maori; Marathi; Maasai; Maldivian/Dhivehi; Mende; Macedonian; Malagasy; Malayalam; Maltese; Malay/Indonesian; Magindanao-Maranao; Mongolian (Khalkha); Manx Gaelic; Moore/Mossi; Mauritian/Seychelles Creole; Munda (Any); Maya (Any); Nahuatl/Mexicano; Nama/Damara; Nubian (Any); Ndebele; Nepali; Norwegian; Nuer/Naadh; Nupe; Newari; Nzema; Ambo/Oshiwambo; Ogoni (Any); Oriya; Oromo; Other Language / Unknown / Unable to communicate; Pangasinan; Pampangan; Pashto/Pakhto; Pahari/Himachali (India); Pahari (Pakistan); Punjabi; Polish; Portuguese; Persian/Farsi; Quechua; Rajasthani/Marwari; Romany/English Romanes; Romani (International); Romanian; Romansch; Runyakitara; Russian; Samoan; Serbian/Croatian/Bosnian; Scots; Shilluk/Cholo; Shona; Sidamo; Sign Language (Other); Slovak; Slovenian; Sindhi; Sango; Sinhala; Somali; Spanish; Sardinian; Siraiki; Sotho/Sesotho; Swazi/Siswati; Tswana/Setswana; Swahili/Kiswahili; Swedish; Tamil; Telugu; Temne; Teso/Ateso; Tigre; Tagalog/Filipino; Tigrinya; Thai; Tibetan; Tiv; Berber/Tamazight; Tonga/Chitonga (Zambia); Tongan (Oceania); Tok Pisin; Traveller Irish/Shelta; Tsonga; Turkmen; Tulu; Tumbuka; Turkish; Ukrainian; Umbundu; Urdu; Urhobo-Isoko; Uyghur; Uzbek; Venda; Vietnamese; Visayan/Bisaya; Wa-Paraok (South-East Asia); West-African Creole Portuguese; Wolof; West-African Pidgin English; Xhosa; Yao/Chiyao (East Africa); Yiddish; Yoruba; Zande; Zulu; Refused.
